# Extracellular vesicles from induced pluripotent stem cell–derived periodontal ligament cells enhance proliferation and migration of periodontal ligament cells for periodontal regeneration

**DOI:** 10.3389/fcell.2026.1821829

**Published:** 2026-08-27

**Authors:** Yurie Taniguchi, Kengo Iwasaki, Tomofumi Yamamoto, Jun-Ichiro Jo, Hiroki Ishikawa, Yoshihiro Momota, Yoshiya Hashimoto

**Affiliations:** 1 Department of Anesthesiology, Graduate School of Dentistry, Osaka Dental University, Hirakata/Osaka, Japan; 2 Division of Creative and Integrated Medicine, Advanced Medicine Research Center, Translational Research Institute for Medical Innovation (TRIMI), Osaka Dental University, Hirakata/Osaka, Japan; 3 Division of Biological Chemistry and Biologicals, National Institute of Health Sciences, Kawasaki, Kanagawa, Japan; 4 Department of Biomaterials, Osaka Dental University, Hirakata/Osaka, Japan; 5 First Department of Oral and Maxillofacial Surgery, Osaka Dental University, Hirakata/Osaka, Japan; 6 Department of Anesthesiology, Osaka, Dental University, Hirakata/Osaka, Japan

**Keywords:** extracellular vesicles, induced pluripotent stem cells, microRNA, paracrine signaling, periodontal ligament cells

## Abstract

**Background:**

Periodontal ligament (PDL) cells play a central role in periodontal tissue regeneration. We previously established induced pluripotent stem cell–derived PDL-like cells (iPS-PDL) as a potential alternative cell source for regenerative therapy. However, the properties and biological functions of extracellular vesicles (EVs) released by iPS-PDL remain unknown. Characterizing these EVs may facilitate the development of cell-free approaches for periodontal regeneration.

**Objective:**

We aimed to isolate and characterize EVs derived from iPS-PDL (iPS-PDL-EVs) and compare their biological functions with those of primary PDL cell–derived EVs (PDL-EVs).

**Methods:**

EVs were isolated and purified using size exclusion chromatography and characterized by nanoparticle tracking analysis, scanning transmission electron microscopy, nano flow cytometry, and Western blotting. Their effects on PDL cell proliferation and migration were evaluated using WST-8 and Transwell assays. miRNA sequencing and bioinformatic analyses were performed to identify differentially expressed miRNAs and enriched biological pathways.

**Results:**

iPS-PDL-EVs and PDL-EVs exhibited typical EV characteristics. Compared with PDL-EVs, iPS-PDL-EVs significantly enhanced PDL cell proliferation and migration and activated MAPK and Akt signaling. Approximately 80% of the identified miRNAs were shared between the two EV populations; however, iPS-PDL-EVs contained elevated levels of miRNAs associated with MAPK signaling. miR-181a-2-3p suppressed *IL1R1*, whereas let-7i-5p and let-7g-5p suppressed *FAS*, thereby promoting PDL cell proliferation and migration.

**Conclusion:**

iPS-PDL-EVs exhibit superior regenerative activity compared with PDL-EVs and may promote periodontal regeneration through miRNA-mediated signaling. These findings support the potential of iPS-PDL-EVs as a cell-free therapeutic strategy for periodontal regeneration. Future *in vivo* studies are required to evaluate their therapeutic efficacy and safety.

## Graphical Abstract

**Figure:**
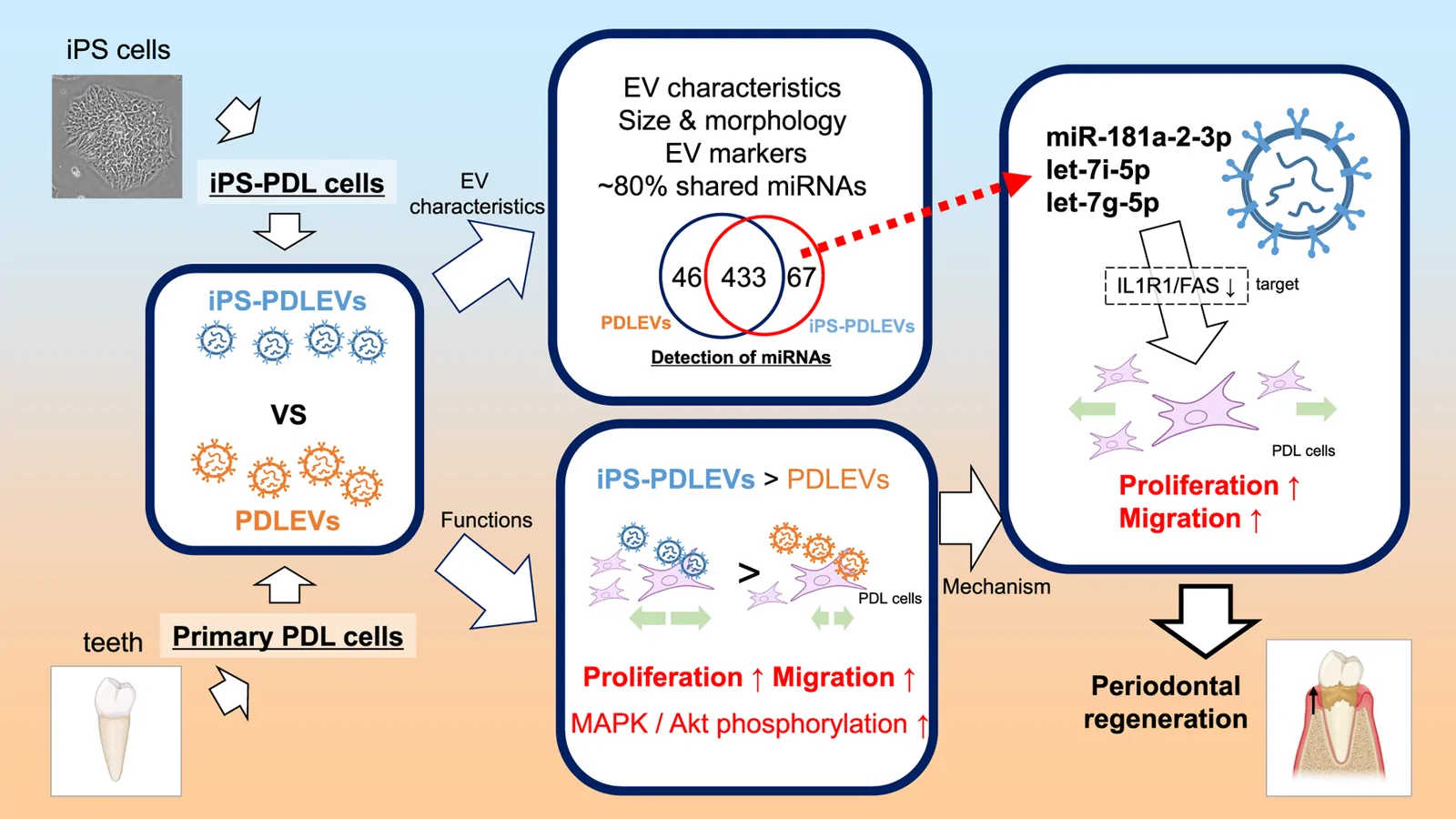

## 1 Introduction

The periodontal ligament (PDL) is a thin, fibrous, soft tissue that connects the tooth root to the alveolar bone (Beertsen et al., 1997). It absorbs occlusal forces through densely arranged collagen fiber bundles, acting as a cushion to protect the tooth from external occlusal forces. Furthermore, PDL serves as a source of progenitor and stem cells that contribute to the maintenance of periodontal tissue homeostasis and wound healing (McCulloch and Bordin, 1991; Melcher, 1976; Polimeni et al., 2006). PDL cells are considered the primary effector cells in periodontal tissue regeneration.

Periodontal disease is a chronic inflammatory disease caused by periodontal pathogens, characterized by the destruction of periodontal tissues (Darveau, 2010; Pihlstrom et al., 2005). Transplantation of PDL cells cultured outside the body induces periodontal regeneration; therefore, PDL cell transplantation has potential in regenerative medicine (Feng et al., 2010; Hasegawa et al., 2005). However, as tooth extraction is required to obtain PDL cells, fundamental constraints exist regarding cell source availability and patient burden (Di Vito et al., 2023; Wen et al., 2024).

Induced pluripotent stem cells (iPSCs) are stem cells that acquire pluripotency by introducing specific genes into somatic cells (Takahashi et al., 2007). They are promising cell sources for regenerative medicine because they can differentiate into nearly all cell types in the body. Clinical trials using iPSC-derived cells are underway for Parkinson’s disease, corneal epithelial stem cell depletion syndrome, age-related macular degeneration, spinal cord injury, and heart failure; therapeutic effects have been reported (Sawamoto et al., 2025; Choi and Liu, 2025; Kirkeby et al., 2025). In our previous study, we reported that soluble factors secreted by PDL cells enabled the efficient differentiation of iPSCs into PDL-like cells (iPS-PDL) (Wu et al., 2025). The resulting iPS-PDL expressed PDL-related genes, such as *ASPN*/*PLAP-1*, *POSTN*, and *COL1A1*, and retained their differentiation potential into osteoblasts and chondrocytes. Furthermore, the transplantation of iPS-PDL into surgically created periodontal defects in rats resulted in the regeneration of periodontal tissue structures comprising cementum, PDL, and the alveolar bone. This finding suggests that iPS-PDL hold promise as a novel cellular source for periodontal regenerative therapy, potentially replacing PDL cells. However, the molecular mechanisms by which the transplanted iPS-PDL induce periodontal tissue regeneration remain unclear.

Extracellular vesicles (EVs) are nanoscale vesicles enclosed by a lipid bilayer membrane containing cargo, such as mRNA, miRNA, DNA, proteins, and metabolites (Théry et al., 2002; Welsh et al., 2024).

EVs mediate intercellular communication through internalization by recipient cells, thereby regulating their function and behavior. Numerous studies in the field of regenerative medicine have demonstrated that EVs secreted by transplanted cells can serve as major paracrine mediators that induce tissue regeneration (Lai et al., 2010; Phinney and Pittenger, 2017). Furthermore, mesenchymal stem cell (MSC)–derived EVs have recently been reported to enhance periodontal tissue regeneration (Huang et al., 2004); EV transplantation is gaining attention as a promising cell-free regenerative strategy that may overcome several limitations associated with stem cell transplantation. However, the properties and functions of EVs released by iPS-PDL have not been reported.

We aimed to comprehensively analyze the miRNAs present in EVs derived from iPS-PDL, compare them with those in EVs derived from primary PDL cells, and elucidate the biological functions of iPS-PDL-derived EVs.

## 2 Methods

### 2.1 Cell culture

Human iPSCs and human PDL cells were purchased from the RIKEN Bioresearch Center (Tsukuba, Japan) and Lonza (Basel, Switzerland), respectively. iPS-PDL cell differentiation was induced using the culture supernatant of PDL cells, as described in our previous report (Wu et al., 2025). Three independently differentiated iPS-PDL lines (iPS-PDL1-3) were used for subsequent experiments. iPS-PDL and PDL cells were cultured in 75 cm^2^ flasks (IWAKI, Shizuoka, Japan) using αMEM (Nacalai Tesque Inc., Kyoto, Japan) containing 15% fetal bovine serum (FBS) (HyClone Laboratories, Logan, UT, United States) and 1% penicillin-streptomycin (Nacalai Tesque Inc.) under a humidified atmosphere of 5% CO_2_ at 37 °C. The medium was changed every 3 days; cells at passages 6–10 were used for subsequent experiments.

### 2.2 Isolation of EVs

Cells were seeded at 1.0 × 10^6^ cells per 75 cm^2^ flask (IWAKI) into two flasks. After 24 h of culture, the medium was replaced with αMEM (Nacalai Tesque Inc.) containing 15% EV-depleted FBS (Biosera, Cholet, France). After 48 h, the culture supernatant was collected, centrifuged at 1,500 × g for 10 min, and concentrated using an Amicon Ultra 100k (Merck, Darmstadt, Germany) (3,000 rpm for 25 min). The culture supernatant was concentrated approximately 60-fold via ultrafiltration. EVs were collected from the concentrated culture supernatant using size exclusion chromatography with a qEV auto-fraction collector and qEV columns (qEV original/35 nm; Izon, Christchurch, New Zealand). The Auto Fraction Collector (Izon) was set to collect 0.5 mL elution fractions. Concentrated culture supernatant (500 µL) was applied to the qEV column; the four fractions confirmed to contain EVs in the previous experiments were collected and used for the study. EV concentration was measured using a Micro BCA Protein Assay Kit (Thermo Fisher Scientific, Waltham, MA, United States). In the subsequent functional assays, the EV dosage was determined based on the protein concentration measured using the BCA assay.

### 2.3 Characterization of EV properties

The size distribution and concentration of EVs were analyzed using a NanoSight NS300 (Malvern Panalytical, United Kingdom) equipped with the Nanoparticle Tracking Analysis software (Sample Assistant Build 3.4.4). The morphology of the EVs was observed using a 30 kV scanning transmission electron microscope (SU9000, Hitachi High-Tech Corporation, Tokyo, Japan) after negative staining with 2% uranyl acetate. The particle concentration, size distribution, and positivity rate of the surface EV markers were characterized using nano flow cytometry (NanoFCM; Flow Nano Analyzer, NanoFCM Inc., Xiamen, Fujian, China). Calibration was performed using fluorescent beads and a silica nanoparticle cocktail according to the manufacturer’s instructions. The samples were diluted with PBS to a suitable particle concentration range. Surface EV markers were stained with FITC-conjugated antibody (ExoBrite™ 490/515, CD9 Flow Antibody (P003-490); CD63 Flow Antibody (P004-490); and CD81 Flow Antibody (P005-490, Biotium). EV marker protein expression (CD9, CD63, and CD81) was also analyzed using Western blotting, whereas negative markers such as calnexin were also evaluated. The protein content of the EVs was measured using a BCA protein assay, adjusted for standardization. The primary antibodies used were mouse monoclonal anti-CD9 (clone 12A12, dilution 1:1000) and anti-CD63 (clone 8A12, dilution 1:1000) from Cosmo Bio (Tokyo, Japan). Calnexin (C5C9, dilution 1:1000) from Cell Signaling Technology (CST), (Danvers, MA, United States). Secondary antibodies (horseradish peroxidase–conjugated anti-mouse IgG, NA931; dilution 1:5000) were purchased from GE Healthcare (Chicago, IL, United States). Equal amounts of protein were loaded into each lane of a Mini-PROTEAN TGX gel (4%–15%, Bio-Rad, Hercules, CA, United States). Antibody signals were detected using chemiluminescence with ImmunoStar LD reagent (290-69904, FUJIFILM Wako Pure Chemical Corporation, Osaka, Japan) and analyzed using a luminescence imaging system (LAS-4000 mini; Fujifilm Corporation, Tokyo, Japan).

### 2.4 Uptake of EVs into PDL cells

EVs were fluorescently labeled using an ExoSparkler Exosome Membrane Labeling Kit (Dojindo, Kumamoto, Japan) according to the manufacturer’s instructions. Fluorescently labeled EVs were added to PDL cells seeded at a concentration of 2.5 × 10^4^ cells per well in a Glass Base Dish (IWAKI) and cultured for 4 h. Next, the cells were fixed in 4% paraformaldehyde-PBS and stained with Hoechst 33342 (Nacalai Tesque Inc.). Fluorescence images of the cells were acquired using a fluorescence microscope (BZ-X800; KEYENCE, Osaka, Japan).

### 2.5 Cell proliferation assay

PDL cells (5.0 × 10^3^ cells) were seeded into a 96-well plate. EV concentrations of 0, 0.25, 0.5, and 1.0 μg/mL were prepared by adjusting the concentration with PBS; the effective concentration of EVs on PDL cells was determined. Next, 10 µL Cell Count Reagent SF (Nacalai Tesque Inc.) was added to each well and incubated at 37 °C for 1 h; subsequently, the absorbance was measured at 450 nm.

### 2.6 Cell migration assay

Transwell inserts (pore size 8.0 µm) (Corning Inc., Corning, NY, United States) were placed onto a 24-well plate. αMEM containing EVs was added to the lower well of the 24-well plate. Next, 5.0 × 10^3^ PDL cells suspended in αMEM containing 0.5% FBS and 1% penicillin-streptomycin were seeded onto the insert. The effective concentrations of EVs were 0, 0.25, 0.5, and 1.0 μg/mL. After 24 h of culture at 37 °C in a 5% CO_2_ incubator, non-migrated cells above the insert were removed with a cotton swab. Cells within the insert were fixed and stained with crystal violet (Nacalai Tesque Inc.); migrating cells were counted using BZ-X800.

### 2.7 Western blotting

Total cellular protein was extracted using RIPA lysis buffer (Nacalai Tesque Inc.). Equal amounts of protein (12 μg per lane) were separated using SDS-PAGE and transferred onto PVDF membranes. The membranes were blocked and incubated with primary antibodies at room temperature for 1 h, followed by horseradish peroxidase–conjugated secondary antibodies at room temperature for 30 min. The following primary antibodies were used: Akt (#4685; Cell Signaling Technology (CST), Danvers, MA, United States), phospho-Akt (CST; #4060), ERK (CST; #4695), phospho-ERK (CST; #4370), p38 MAPK (CST; #8690), phospho-p38 MAPK (CST; #4511), JNK (#9252), and phospho-JNK (CST; #4668, #4970). A horseradish peroxidase–conjugated anti-rabbit IgG antibody (CST; #7074) was used as the secondary antibody. β-Actin was used as the loading control. All antibodies were diluted 1:1000. Signals were visualized with ImmunoStar Zeta (Wako, Osaka, Japan) with a 5-min exposure and detected using the ChemiDoc imaging system (Bio-Rad).

### 2.8 Comprehensive miRNA sequencing in EVs

Total RNA was extracted from EVs using a microRNA Extractor Kit for EVs (FUJIFILM Wako Pure Chemical Corporation). Small RNA libraries were prepared via 30 and 50 adapter ligations, reverse transcription, and PCR amplification using Illumina’s NEB Next Multiplex Small RNA Library Prep Set (New England Biolabs, Inc., Ipswich, MA, United States) according to the manufacturer’s protocol. NGS was performed under Illumina 100 bp single-read conditions. Raw data read count normalization and differential expression analyses were performed using the trimmed mean of the M-values method and edgeR (version 3.26.8), respectively. Comprehensive miRNA sequencing was performed according to the manufacturer’s protocol. miRNAs that were highly expressed in iPS-PDL were selected (p < 0.05, mean count > 1000, and (PDL cells/iPS-PDL) < 0.75). We used TargetScan (https://www.targetscan.org/vert_80/) to perform target screening. Additionally, we used the RNA sequencing data of iPS-PDL and PDL cells from our previous report (Wu et al., 2025). RNA sequencing data were submitted to the DDBJ Sequence Read Archive (Bio Project accession number: PRJDB39888, DRA Run accession number: DRR913328-DRR913336). Gene Set Enrichment Analysis (GSEA) of these data was performed (https://www.gsea-msigdb.org/gsea/index.jsp) (p < 0.05 and Fold change > ±2).

### 2.9 miRNA transfection

PDL cells (1.0 × 10^5^ cells) were seeded and cultured in αMEM (Nacalai Tesque Inc.) containing 10% FBS and 1% penicillin-streptomycin at 37 °C. Upon reaching 50% confluence, cells were washed with PBS(−) and transfected with the target miRNAs (hsa-miR-181a-2-3p, hsa-let-7i-5p, and hsa-let-7g-5p) and a control miRNA (negative control [NC]: hsa-miR-181nega) (KOKEN, Tokyo, Japan), which were introduced at 20 nM each using RNAiMAX (3.0 µL/well) (Thermo Fisher Scientific) and Opti-MEM (Gibco, Thermo Fisher Scientific).

### 2.10 Gene expression analysis using quantitative reverse transcription PCR (qRT-PCR)

Total RNA was extracted using an RNeasy Mini Kit (QIAGEN, Venlo, Netherlands); cDNA was synthesized using ReverTra Ace qPCR RT Master Mix (TOYOBO, Osaka, Japan). qRT-PCR was performed using THUNDERBIRD Next SYBR™ qPCR Mix (TOYOBO) and the QuantStudio 3 Real-Time PCR System (Thermo Fisher Scientific). Gene expression levels were calculated using the ΔΔCT method, with GAPDH as the internal control. The following primer sequences were used: GAPDH: forward, 5ʹ-GGA​GCG​AGA​TCC​CTC​CAA​AAT-3ʹ; reverse, 5ʹ-GGC​TGT​TGT​CAT​ACT​TCT​CAT​GG-3ʹ; Fas: forward, 5ʹ-GGA​CCC​AGA​ATA​CCA​AGT​GCA​G-3ʹ; reverse, 5ʹ-GTT​GCT​GGT​GAG​TGT​GCA​TTC​C-3ʹ; IL1R1: forward, 5ʹ-GTG​CTT​TGG​TAC​AGG​GAT​TCC​TG-3ʹ; reverse, 5ʹ-CAC​AGT​CAG​AGG​TAG​ACC​CTT​C-3ʹ.

### 2.11 Cell proliferation with miRNA mimic transfection

Cell proliferation was assessed using Cell Count Reagent SF (Nacalai Tesque Inc.). The cells were seeded in a 96-well plate at a density of 3 × 10^3^ cells/well. After specific incubation times following the addition of miRNA mimics or EVs, CCK-8 reagent was added; the plate was incubated at 37 °C for 1 h. The absorbance was measured at 450 nm; the cell proliferation rate per well was calculated based on this value.

### 2.12 Cell migration with miRNA mimic transfection

To evaluate the effects of miRNA transfection on cell migration, Transwell inserts (pore size 8.0 µm) (Corning Incorporated, New York, United States) were placed; PDL cells (5.0 × 10^3^ cells) were seeded onto the upper chamber using αMEM containing 10% FBS (Nacalai Tesque Inc.). miRNA transfection was performed using RNAiMAX (Thermo Fisher Scientific) and Opti-MEM (Gibco, Thermo Fisher Scientific). Additionally, αMEM containing 10% FBS (Nacalai Tesque Inc.) was added to the lower well. After 24 h of culture at 37 °C in a 5% CO_2_ incubator, the non-migrating cells on the upper insert were removed using a cotton swab. The cells within the insert were fixed, stained with crystal violet (Nacalai Tesque Inc.), and photographed under a fluorescence microscope (BZ-X800). The cells were counted using the BZ-X800 Analyzer.

### 2.13 Statistical analysis

Normality and homogeneity of variance were assessed using the Brown–Forsythe test, respectively. All experiments were conducted independently three times using biological replicates. The results are presented as mean ± standard deviation. Comparisons between two groups were analyzed using Student’s t-test. For comparisons among more than two groups, one-way ANOVA was performed, followed by Tukey’s post-hoc honest significant difference test to determine which groups differed from each other. All statistical analyses were performed using GraphPad Prism 10.3.0. Differences were considered statistically significant at P < 0.05.

## 3 Results

### 3.1 Characterization of EVs

**FIGURE 1:**
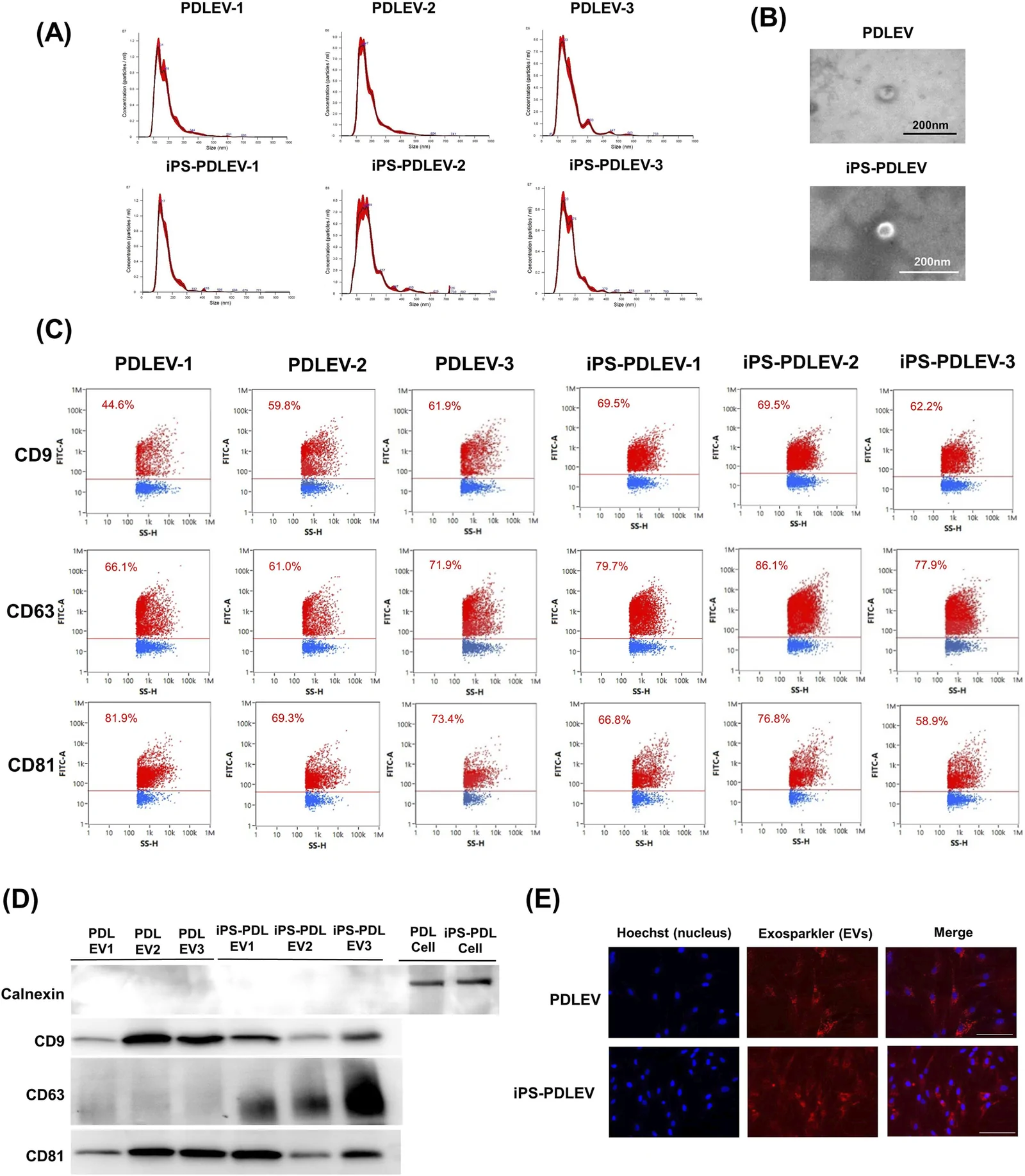
Characterization of PDL-EVs and iPS-PDL-EVs. **(A)** Nanoparticle tracking analysis of PDL-EVs and iPS-PDL-EVs. Both EV populations exhibited a size distribution with a peak at approximately 140 nm. **(B)** Scanning transmission electron microscopy images of PDL-EVs and iPS-PDL-EVs showing typical EV-like morphology (scale bar: 200 nm). **(C)** NanoFCM analysis of PDL-EVs and iPS-PDL-EVs. EVs positive for the surface markers CD9, CD63, and CD81 are shown in red, whereas negative populations are shown in blue. **(D)** Western blotting showed the expression of EV surface markers and a non-EV marker (Calnexin) was only in cells and was not detected in EVs. PDL-EVs and iPS-PDL-EVs derived from three independent PDL cell batches (PDL1–3) and iPS-PDL cell batches (iPS-PDL1–3) expressed CD9, CD63, and CD81. **(E)** Uptake of EVs by PDL cells. Fluorescently labeled PDL-EVs and iPS-PDL-EVs were detected within the cytoplasm of PDL cells, indicating efficient internalization (scale bar: 100 µm).

EVs were first recovered from the culture supernatants of iPS-PDL and primary PDL cells. Particle size analysis of EVs obtained from iPS-PDL and primary PDL cells using nanoparticle tracking analysis revealed a distribution peaking at 110–160 nm, with particle concentrations ranging from 1 × 10^8^ to 1 × 10^9^ particles/mL (Figure 1A). Observation of the structure of iPS-PDL-EVs and PDL-EVs using scanning transmission electron microscopy confirmed typical EV morphology with diameters less than 200 nm (Figure 1B). Furthermore, analysis of antigen expression on the EV surface using NanoFCM revealed the expression of CD9, CD63, and CD81 in iPS-PDL-EVs and PDL-EVs (Figure 1C). Western blotting showed the expression of EV surface markers and a non-EV marker (Calnexin) was only in cells and was not detected in EVs (Figure 1D). Original Western blot membrane images are shown in Supplementary Figure S1. These results confirmed that EVs were recovered from iPS-PDL and primary PDL cells.

### 3.2 Uptake of EVs into PDL cells

PDL cells play a crucial role in periodontal tissue regeneration; their proliferation and migration toward wound sites are essential processes in this context (McCulloch and Bordin, 1991; Melcher, 1976). Based on this rationale, we used PDL cells as recipient cells to evaluate the effects of iPS-PDL-EVs and PDL-EVs. To confirm the uptake of each EV type into PDL cells, iPS-PDL-EVs and PDL-EVs were fluorescently labeled using ExoSparkler and incubated with PDL cells. As shown in Figure 1E, fluorescence imaging demonstrated the uptake of both EV types into the cytoplasm of PDL cells.

### 3.3 Effects of EVs on the proliferation and migration of PDL cells

**FIGURE 2:**
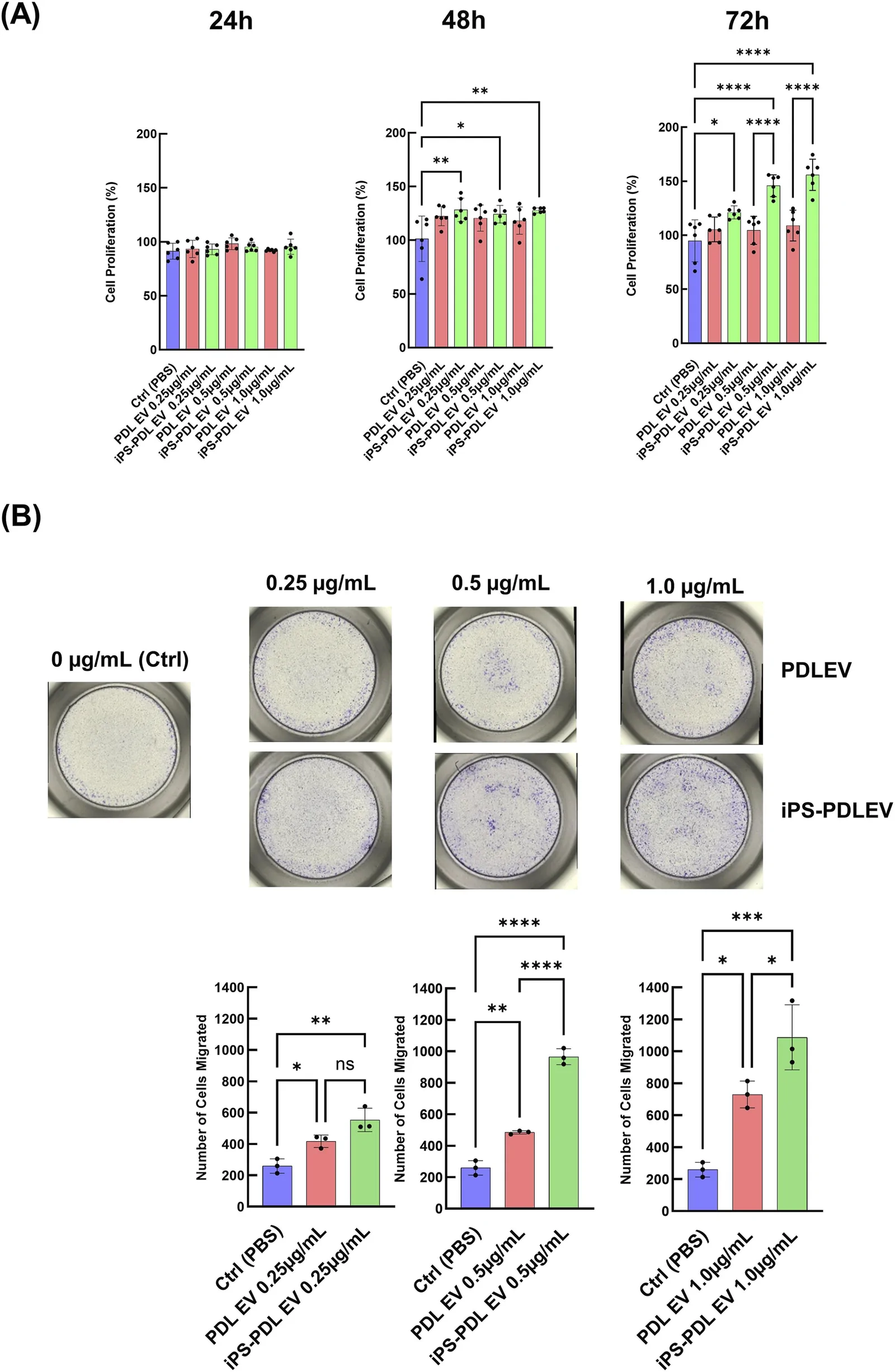
Effects of PDL-EVs and iPS-PDL-EVs on proliferation and migration of PDL cells. **(A)** Effects of PDL-EVs and iPS-PDL-EVs on PDL cell viability at 24, 48, and 72 h, assessed using the WST-8 assay. iPS-PDL-EVs significantly enhanced PDL cell proliferation at 48 and 72 h, whereas PDL-EVs did not show a significant proliferative effect compared with the control. **(B)** Effects of different concentrations of PDL-EVs and iPS-PDL-EVs on PDL cell migration, evaluated using a Transwell migration assay. Representative images and quantitative analysis of migrated cells are shown. Both EVs promoted PDL cell migration, whereas iPS-PDL-EVs significantly increased the number of migrated cells at 0.5 and 1.0 μg/mL compared with PDL-EVs (scale bar: 100 µm). Data are presented as mean ± SD. *P < 0.05, **P < 0.01, ***P < 0.001, ****P < 0.0001. The significance bars indicate the specific pairwise comparisons shown in each graph (PBS control vs. EV-treated groups and PDL-EV vs. iPS-PDL-EV groups).

We examined the effects of iPS-PDL-EVs and PDL-EVs on PDL cell proliferation. The results showed that at 48 and 72 h post-addition, iPS-PDL-EVs exhibited a significant cell proliferation-promoting effect at all concentrations compared with the control group (PBS) (Figure 2A). In contrast, PDL-EVs showed no significant changes compared with the control at any time point (*P < 0.05, **P < 0.01, ***P < 0.001, ****P < 0.0001). The effects of iPS-PDL-EVs and PDL-EVs on PDL cell migration were examined using a Transwell system. After 24 h, the number of PDL cells that migrated through the membrane with 8-µm pores was measured. The results showed that at all concentrations (0.25, 0.5, and 1.0 μg/mL), iPS-PDL-EVs and PDL-EVs significantly increased the number of migrating cells compared with the control group (PBS) (Figure 2B). Comparison of the effects of iPS-PDL-EVs and PDL-EVs showed that iPS-PDL-EVs induced cell migration significantly more than PDL-EVs at 0.5 and 1.0 μg/mL. However, no difference was observed between the two groups at 0.25 μg/mL (*P < 0.05, **P < 0.01, ***P < 0.001, ****P < 0.0001).

### 3.4 Phosphorylation of p38, JNK, ERK, and Akt signals in PDL cells treated with iPS-PDL-EVs

**FIGURE 3:**
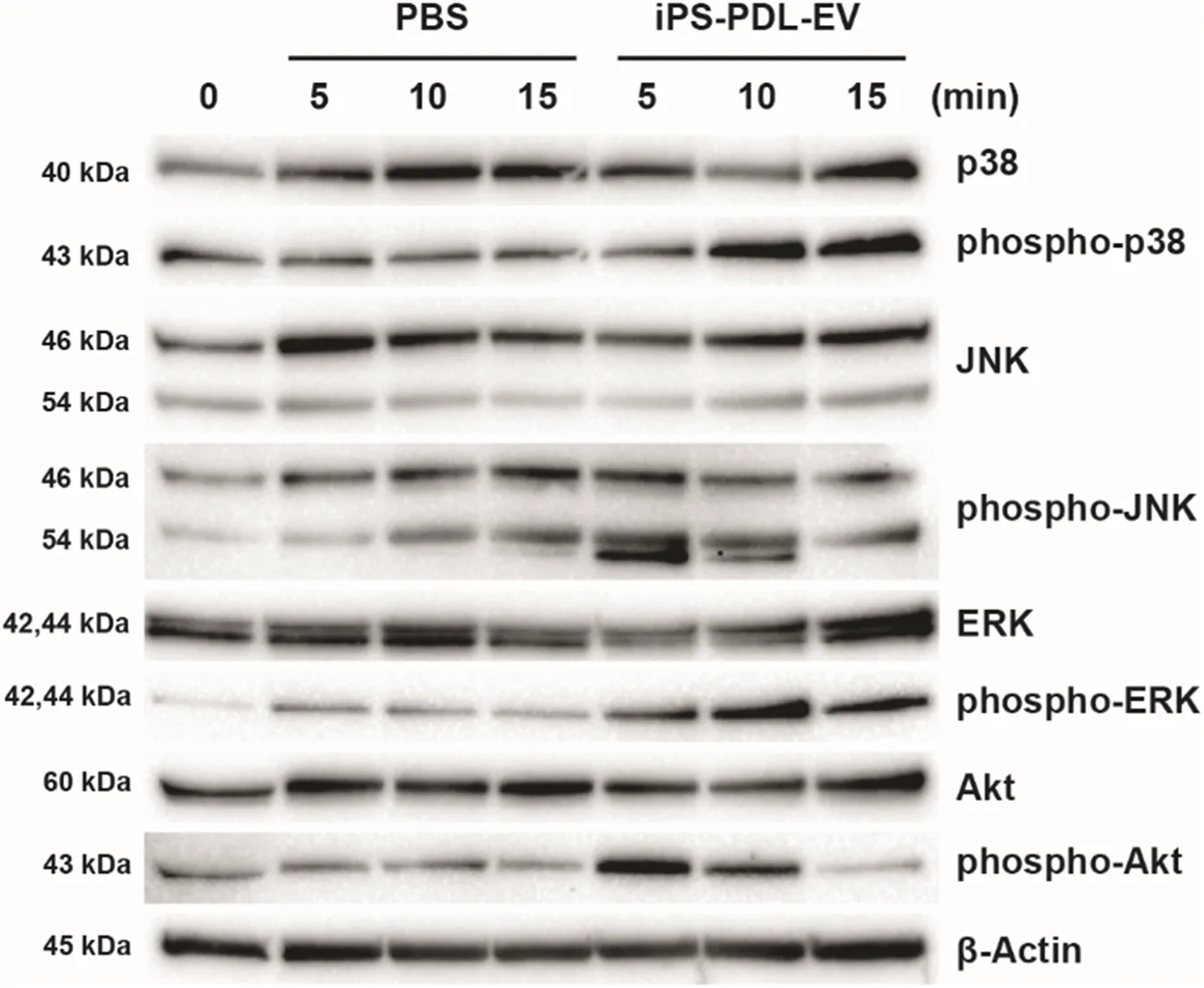
Activation of MAPK and Akt signaling pathways by iPS-PDL-EVs. Representative Western blot images showing the phosphorylation of MAPK family members (p38, JNK, and ERK) and Akt in PDL cells are presented. Treatment with iPS-PDL-EVs markedly increased the phosphorylation levels of p38, JNK, ERK, and Akt compared with control conditions (PBS). β-Actin was used as the loading control.

The MAPK and Akt signaling pathways play major roles in regulating cell proliferation and migration (Huang et al., 2004; Manning and Toker, 2017). Therefore, we examined the phosphorylation status of MAPK (p38, JNK, and ERK) and Akt in PDL cells treated with iPS-PDL-EVs. As shown in Figure 3, treatment of PDL cells with iPS-PDL-EVs resulted in an increased phosphorylation of p38, JNK, ERK, and Akt within 5–15 min. Original Western blot membrane images are shown in Supplementary Figure S2.

### 3.5 Comparison of signaling pathway activity between iPS-PDL and PDL cells using GSEA

**FIGURE 4:**
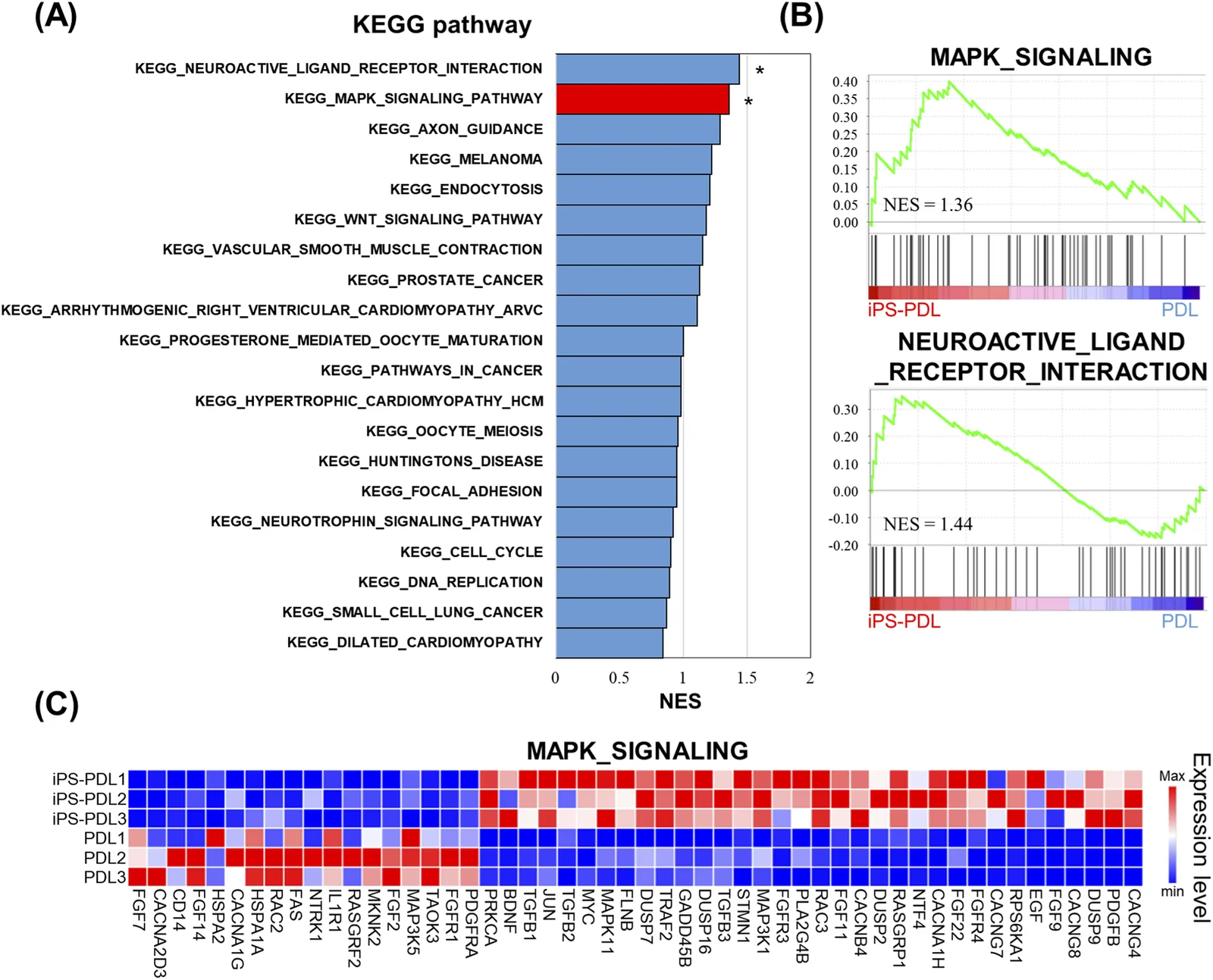
GSEA comparing iPS-PDL and primary PDL cells. **(A)** Bar plot showing normalized enrichment scores for selected KEGG pathways. **(B)** Enrichment plots for the MAPK signaling and neuroactive ligand–receptor interaction pathways. **(C)** Heat map of leading-edge genes from the MAPK signaling pathway, showing gene-wise Z-scored expression levels across biological replicates. Genes associated with the MAPK signaling pathway were highly enriched and upregulated in iPS-PDL compared with those in primary PDL cells.

To analyze the differences in the transcriptional profiles of parental cells (iPS-PDL and PDL cells) at the pathway level, GSEA was performed using KEGG pathway analysis. The results showed consistent enrichment in iPS-PDL for pathways such as MAPK signaling and neuroactive ligand–receptor interactions (Figure 4A). The enrichment plot showed that the gene groups belonging to these pathways were distributed with a bias toward iPS-PDL (Figure 4B). Furthermore, heatmap analysis using leading-edge genes related to MAPK signaling revealed common expression trends across biological replicates, indicating differences in the transcriptional regulation of MAPK signaling between the two cell types (Figure 4C).

### 3.6 Comprehensive sequence analysis of miRNAs contained in iPS-PDL-EVs and PDL-EVs

**FIGURE 5:**
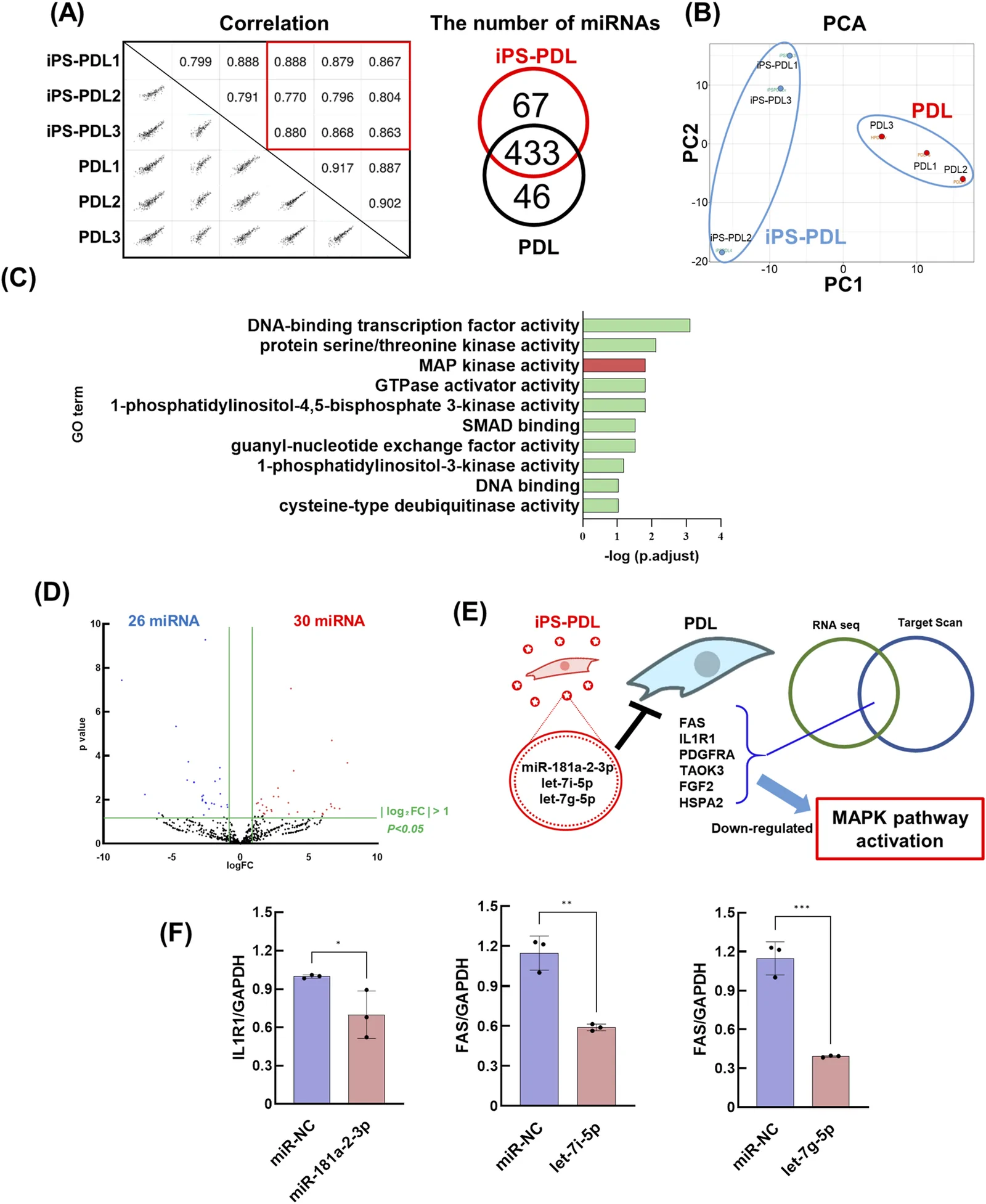
Comparison of miRNA contents between iPS-PDL-EVs and PDL-EVs analyzed by miRNA sequencing. **(A)** Correlation heatmap of miRNA expression profiles in iPS-PDL-EVs and PDL-EVs (left panel) and the number of detected miRNAs in iPS-PDL-EVs and PDL-EVs (right panel). **(B)** Principal component analysis of miRNA expression profiles in iPS-PDL-EVs and PDL-EVs. **(C)** Gene Ontology annotation of molecular function terms associated with differentially expressed miRNAs. **(D)** Volcano plot showing differentially expressed miRNAs between iPS-PDL-EVs and PDL-EVs. miRNAs with significant differences (fold change > 1.0, P < 0.05) are highlighted in red (enriched in iPS-PDL-EVs) and blue (enriched in PDL-EVs). **(E)** Schematic illustration of the target screening strategy for miRNAs upregulated in iPS-PDL-EVs. **(F)** qRT–PCR analysis of candidate gene expression in PDL cells 24 h after transfection with representative miRNA mimics. miR-NC was used as the negative control. Data are presented as mean ± SD (n = 3). *P < 0.05, **P < 0.01, ***P < 0.001 versus the miR-NC group.

Subsequently, we compared the miRNAs contained in iPS-PDL-EVs and PDL-EVs using miRNA sequencing. A correlation diagram for the miRNAs in iPS-PDL-EVs and PDL-EVs is shown in Figure 5A. The correlation coefficients between iPS-PDL-EVs and PDL-EVs were very high, suggesting that the miRNAs contained in the EVs exhibited high similarity. Furthermore, 500 and 479 distinct miRNAs were detected in iPS-PDL-EVs and PDL-EVs, respectively, with 433 miRNAs being commonly detected in both EV types (Figure 5A). These results indicated that approximately 80% of the miRNAs contained in iPS-PDL-EVs and PDL-EVs were common, demonstrating the high similarity between the EVs. However, regarding differentially expressed miRNAs, 67 miRNAs were specifically detected in iPS-PDL-EVs, whereas 46 specific miRNAs were identified in PDL-EVs. Principal component analysis showed that biological replicates of iPS-PDL-EVs and PDL-EVs were tightly clustered within each group, whereas the two groups were clearly separated (Figure 5B). Gene Ontology analysis revealed their involvement in diverse biological pathways, including those related to MAPK signaling (Figure 5C). Furthermore, in iPS-PDL-EVs, 30 and 26 miRNAs were significantly upregulated and downregulated, respectively (Figure 5D). Among the miRNAs specifically contained in iPS-PDL-EVs with significant differences in expression intensity (p < 0.05, signal value > 1000, PDL/iPS < 0.75), miR-181a-2-3p, let-7i-5p, and let-7g-5p were selected for further investigation (Figure 5E).

### 3.7 Suppression of PDL gene expression by miR-181a-2-3p, let-7i-5p, and let-7g-5p

TargetScan analysis of the candidate genes of the three selected miRNAs revealed that *FAS* was a candidate target of let-7i-5p and let-7g-5p, whereas *IL1R1* was a candidate target of miR-181a-2-3p (Figure 5E). The *FAS* and *IL1R1* genes are also recognized as genes regulating MAPK signaling, which is expressed at lower levels in iPS-PDL than in PDL cells (Figure 4C). To examine the suppressive effects of these miRNAs on gene expression in PDL cells, the cells were transfected with let-7i-5p, let-7g-5p, and miR-181a-2-3p mimics. This approach confirmed a decrease in *FAS* expression by the let-7i-5p and let-7g-5p mimics and a decrease in *IL1R1* expression by the miR-181a-2-3p mimic (Figure 5F).

### 3.8 Regulation of PDL proliferation and migration activity by miR-181a-2-3p, let-7i-5p, and let-7g-5p

**FIGURE 6:**
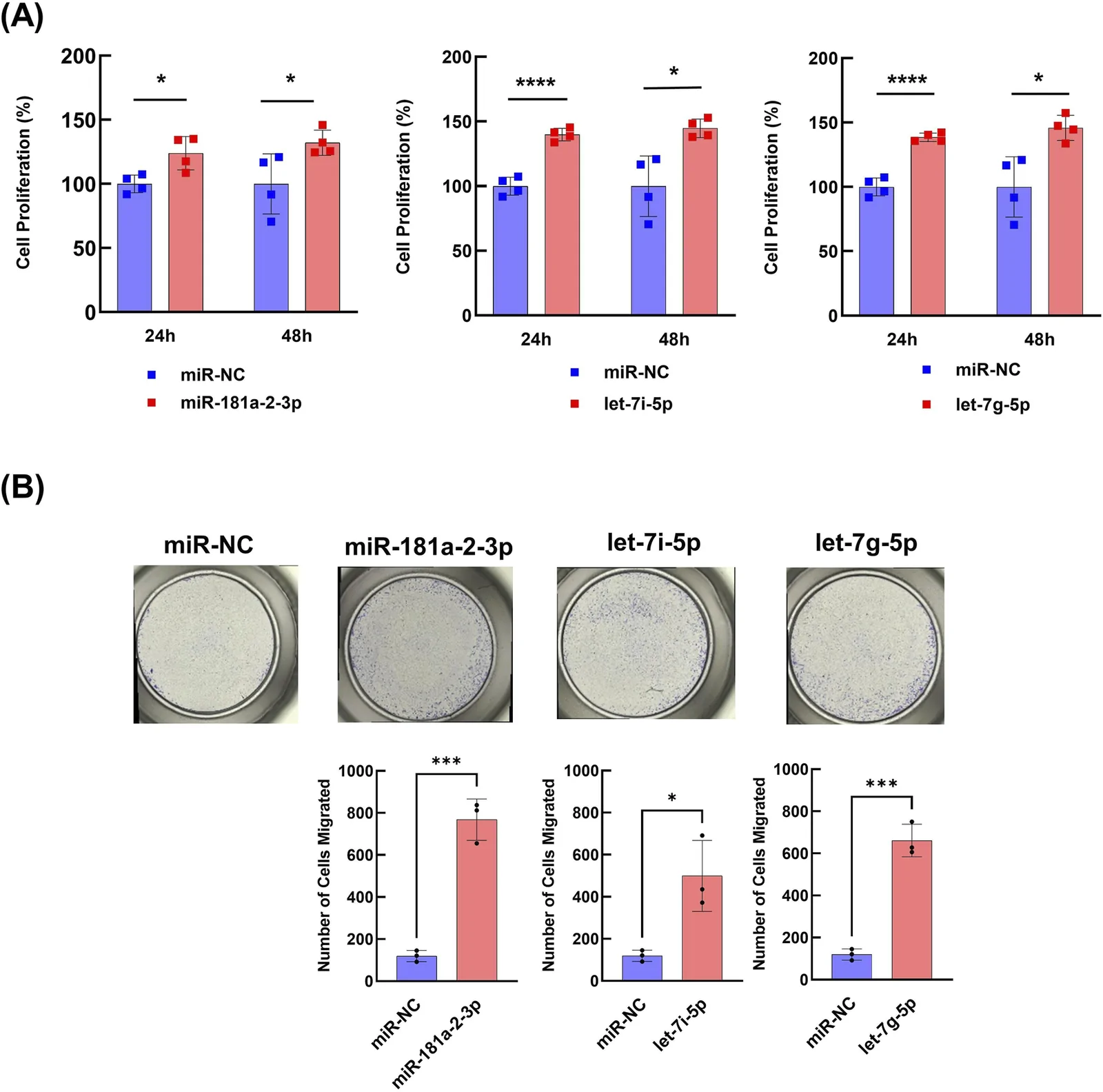
Effects of miRNA mimics on proliferation and migration of PDL cells. **(A)** Cell proliferation assessed using the WST-8 assay following transfection of miRNA mimics. Transfection of miR-181a-2-3p, let-7i-5p, and let-7g-5p significantly increased PDL cell proliferation compared with the negative control at 24 and 48 h. **(B)** Cell migration assessed by a Transwell migration assay following transfection of miRNA mimics. Representative images of migrated cells and quantitative analysis are shown. Transfection of miR-181a-2-3p, let-7i-5p, and let-7g-5p significantly increased the number of migrated PDL cells compared with the negative control. Data are presented as mean ± SD. *P < 0.05, ***P < 0.001, ****P < 0.0001 vs. miR-NC.

The effects of let-7i-5p, let-7g-5p, and miR-181a-2-3p on PDL cell proliferation and migration were investigated. We introduced let-7i-5p, let-7g-5p, and miR-181a-2-3p mimics into PDL cells and observed the changes in cell proliferation and migration. As shown in Figure 6A, let-7i-5p, let-7g-5p, and miR-181a-2-3p mimics significantly enhanced PDL cell proliferation. Furthermore, as shown in Figure 6B, let-7i-5p, let-7g-5p, and miR-181a-2-3p mimics significantly promoted PDL cell migration, suggesting a regulatory role for these miRNAs in PDL cell proliferation and migration.

## 4 Discussion

In this study, we compared the functions of EVs derived from iPS-PDL with those of EVs derived from primary PDL cells, focusing on their effects on PDL cell proliferation and migration. The results revealed that iPS-PDL-EVs significantly promoted PDL cell proliferation and migration compared with PDL-EVs. Furthermore, we found that iPS-PDL-EVs rapidly activated MAPK signaling in PDL cells. Additionally, iPS-PDL-EVs contained significantly higher levels of miR-181a-2-3p, let-7i-5p, and let-7g-5p than PDL-EVs, which are involved in the regulation of MAPK signaling. The introduction of these miRNAs into PDL cells significantly enhanced cell proliferation and migration, suggesting that iPS-PDL-EVs increased PDL cell proliferation and migration through these miRNAs. Enhanced PDL cell proliferation and migration are crucial for the regeneration of periodontal tissues (Melcher, 1976; Polimeni et al., 2006). The results of this study may elucidate the molecular basis underlying our previous observations of periodontal tissue regeneration following iPS-PDL transplantation (Wu et al., 2025). Furthermore, these findings suggest the potential of iPS-PDL-EVs for periodontal regenerative therapy. To the best of our knowledge, this is the first detailed analysis of the functions of EVs released by iPS-PDL.

In the present study, iPS-PDL-EVs significantly promoted the proliferation and migration of PDL cells, even at relatively low concentrations ranging from 0.25 to 1 μg/mL. Previous studies using EVs derived from MSCs on PDL cells have reported that EV concentrations of 200 μg/mL and 5 μg/mL enhance cell proliferation and migration (Chew et al., 2019; Liu et al., 2021). Furthermore, when PDL-EVs were applied to bone marrow–derived MSCs, 1 μg/mL of EVs promoted proliferation and migration (Zhao et al., 2022). Compared with these previous EVs, iPS-PDL-EVs are considered to possess higher biological activity, promoting PDL cell proliferation and migration at lower doses. The molecular mechanisms underlying the strong biological effects of iPS-PDL-EVs at low concentrations have not been fully elucidated. However, considering that the properties and contents of EVs strongly reflect the biological characteristics of the parental cells, the higher proliferative capacity of iPS-PDL compared with that of primary PDL cells may be reflected (Li et al., 2026) in the miRNA composition and functional characteristics of the related EVs. However, further investigation into this hypothesis is required.

In this study, we found that PDL cell treatment with iPS-PDL-EVs rapidly activated MAPK and Akt signaling. Furthermore, miRNA sequencing analysis of iPS-PDL-EVs revealed that MAPK activity ranked highly among the biological processes regulated by miRNAs expressed at high levels in iPS-PDL-EVs. These results suggest that iPS-PDL-EVs, once internalized by cells, enhance MAPK signaling via miR-181a-2-3p, let-7i-5p, and let-7g-5p, thereby promoting PDL cell proliferation and migration. iPSC-derived cells abundantly contain miRNAs involved in cell proliferation, migration, and anti-inflammatory responses, such as the let-7 family, miR-21, and miR-10 family (Bai et al., 2022). In this study, the synergistic suppression of *FAS* by let-7g-5p and let-7i-5p, along with the suppression of *IL1R1* by miR-181a-2-3p, may have reduced the susceptibility of PDL cells to apoptosis and inflammatory signaling, ultimately enhancing cell survival and motility. Reduced *FAS* expression decreases susceptibility to cell death (Hau et al., 2012), whereas decreased *IL1R1* suppresses inflammatory signaling via NF-κB and p38 MAPK, thereby alleviating the inflammatory stress that inhibits cell migration (Weber et al., 2010; Nirenjen et al., 2023). RNA sequencing analysis of the parental cells of iPS-PDL-EVs and PDL-EVs revealed that *FAS* and *IL1R1* were reduced in iPS-PDL; MAPK was one of the signaling pathways specifically enriched in iPS-PDL. These findings suggest that enhanced MAPK signaling mediated by miRNAs in iPS-PDL-EVs can explain the high proliferative capacity of iPS-PDL.

Wound healing in periodontal tissues is highly dependent on the cell types that proliferate and migrate to the wound site. True periodontal tissue regeneration occurs only when cells derived from PDL predominantly proliferate and migrate to the wound site (Melcher, 1976; Polimeni et al., 2006). Furthermore, this concept is clinically supported by the fact that guided tissue regeneration techniques utilize membranes as physical barriers to promote PDL cell proliferation and migration and induce periodontal tissue regeneration (Nyman et al., 1982; Gottlow et al., 1986). Therefore, the PDL cell proliferation- and migration-promoting effects of iPS-PDL-EVs suggest their potential for periodontal tissue regeneration. Additionally, a recent report demonstrated that the administration of EVs derived from MSCs induced periodontal tissue regeneration (Chew et al., 2019). Cell-free therapies using EVs are gaining attention as novel regenerative strategies for periodontal disease. Although our previous findings have shown that iPS-PDL cell transplantation induces periodontal tissue regeneration (Wu et al., 2025), the detailed regenerative mechanism remains unclear. These results suggest that transplanted iPS-PDL contribute to tissue regeneration by locally secreting EVs that promote the proliferation and migration of host PDL cells. Future studies are required to verify the periodontal tissue-regenerative effects of iPS-PDL-EVs using a rat periodontal defect model.

This study has some limitations. First, the biological effects of iPS-PDL-EVs were evaluated only *in vitro* using primary human PDL cells. Therefore, whether these findings can be translated into enhanced periodontal tissue regeneration *in vivo* remains to be determined. Additionally, although several regenerative miRNAs and their associated candidate genes were identified, the underlying molecular mechanisms were not fully elucidated. Future studies using animal models of periodontal defects will be necessary to evaluate the therapeutic efficacy, optimal dosage, biodistribution, and long-term safety of iPS-PDL-EVs before clinical application.

## 5 Conclusion

Our results demonstrated that EVs derived from iPS-PDL possessed greater pro-proliferative and pro-migratory effects than those derived from primary PDL cells with respect to inducing cell proliferation and migration. iPS-PDL-EVs efficiently activate intracellular responses, particularly those centered on MAPK signaling, even at low concentrations; they can induce regeneration-related functions in PDL cells. Furthermore, the findings of this study partially elucidate the molecular basis of the periodontal tissue regenerative effects of iPS-PDL transplantation, suggesting that EV-mediated paracrine functions play a crucial role in periodontal tissue regeneration. Therefore, iPS-PDL-EVs may represent promising candidates as cell-free therapeutic strategies for periodontal tissue regeneration.

## Data Availability

The datasets presented in this study can be found in online repositories. The names of the repository/repositories and accession number(s) can be found in the article/Supplementary Material.
