# Supplementary material for: Extracellular vesicles from induced pluripotent stem cell–derived periodontal ligament cells enhance proliferation and migration of periodontal ligament cells for periodontal regeneration
Source: Front Cell Dev Biol. 2026 Aug 27;14:1821829. doi: 10.3389/fcell.2026.1821829 (PMC13558162; doi:10.3389/fcell.2026.1821829)
Supplement: Supplementary file 1 [file DataSheet1.pdf]

Supplementary Figures

Extracellular Vesicles from Induced Pluripotent Stem Cell-Derived Periodontal Ligament Cells Enhance Proliferation and Migration of Periodontal Ligament Cells For Periodontal Regeneration

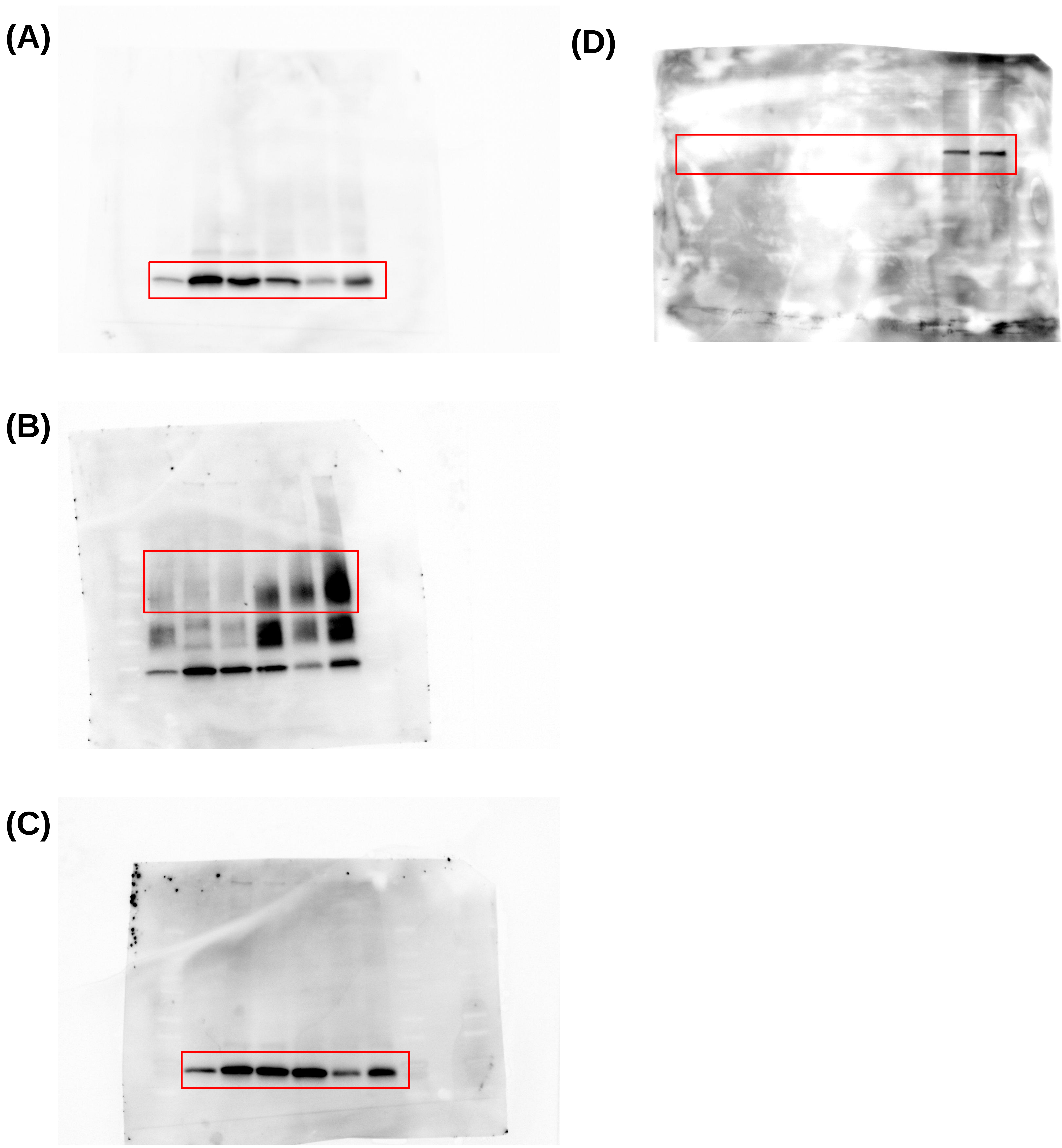

Supplementary Figure 1. Original uncropped images of the Western blots shown in Figure 1D. The original membrane images for CD9 (A), CD63 (B), CD81(C), and calnexin (D) are presented. The red boxes indicate the regions cropped and shown in Figure 1D.

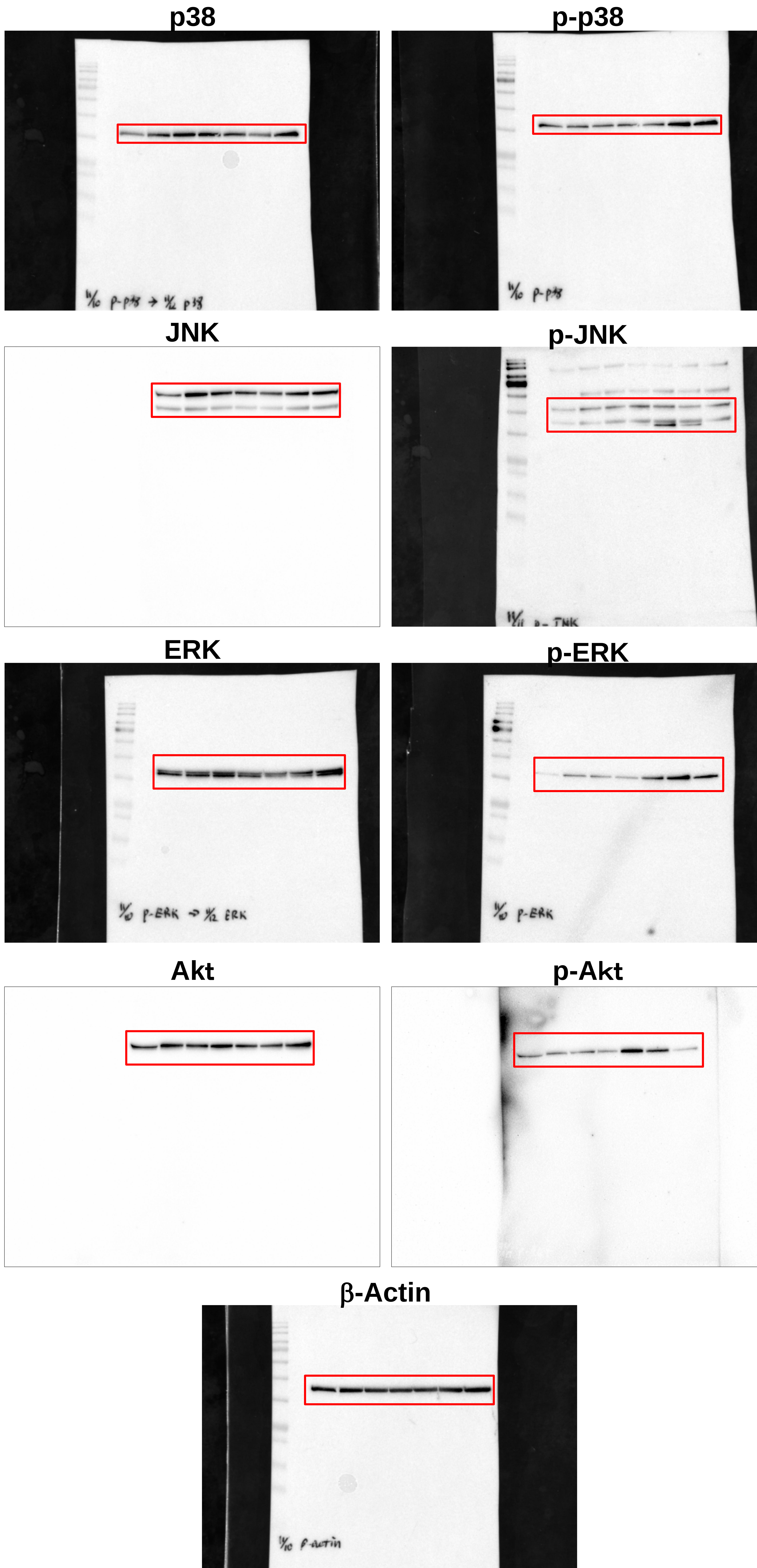

Supplementary Figure 2. Original uncropped images of the Western blots shown in Figure 3. The original membrane images for p38, p-p38, JNK, p-JNK, ERK, p-ERK, Akt, p-Akt, and β–Actin are presented. The red boxes indicate the regions cropped and shown in Figure 3.
